# Antioxidative and anticancer effects of *Tacca chantrieri* extract enhancing cisplatin sensitivity in cholangiocarcinoma cells

**DOI:** 10.1371/journal.pone.0317111

**Published:** 2025-01-16

**Authors:** Napat Armartmuntree, Yingpinyapat Kittirat, Bundit Promraksa, Watcharin Loilome, Hasaya Dokduang, Anchalee Techasen, Pahol Sansomchai, Malinee Thanee, Thomas O’Connor, Phutthida Kongthitilerd, Sureerat Padthaisong

**Affiliations:** 1 Department of Medical Science, Amnatcharoen Campus, Mahidol University, Amnat Charoen, Thailand; 2 Department of Medical Sciences, Regional Medical Sciences Center 2 Phitsanulok, Ministry of Public Health, Mueang District, Phitsanulok Province, Thailand; 3 Department of Systems Biosciences and Computational Medicine, Faculty of Medicine, Khon Kaen University, Khon Kaen, Thailand; 4 Cholangiocarcinoma Research Institute (CARI), Khon Kaen University, Khon Kaen, Thailand; 5 Faculty of Medicine, Mahasarakham University, Mahasarakham, Thailand; 6 Faculty of Associated Medical Sciences, Khon Kaen University, Khon Kaen, Thailand; 7 D De Dee Cosmetic Factory, Lamphun, Thailand; 8 Department of Pathology, Faculty of Medicine, Khon Kaen University, Khon Kaen, Thailand; 9 School of Physiology, Pharmacology and Neuroscience, Bristol University, Bristol, United Kingdom; 10 Faculty of Allied Health Sciences, Burapha University, Chonburi, Thailand; The Hormel Institute (University of Minnesota), UNITED STATES OF AMERICA

## Abstract

Cholangiocarcinoma (CCA) poses a significant healthcare challenge due to the limited effects of chemotherapeutic drugs. Natural products have gained widespread attention in cancer research according to their promising anti-cancer effects with minimal adverse side effects. This study explored the potential of *Tacca chantrieri* (TC), a plant rich in bioactive compounds, as a therapeutic agent for CCA. TC, a traditional remedy in Southeast Asia, exhibits anti-inflammatory and cytotoxic properties against cancer cells. Ethanol extraction of TC’s rhizome was conducted, and antioxidant activities were assessed through various assays, including total phenolic and flavonoid contents, DPPH radical scavenging, and FRAP assays. The cytotoxic effects of TC extracts on CCA cell lines (KKU-213A and KKU-213C) were evaluated using MTT assays and flow cytometry. Protein levels of Bax and Bcl-2 were determined through western blot analysis. Additionally, the study investigated whether the combined impact of TC extract and cisplatin on CCA cells enhanced cisplatin’s efficacy as an anti-cancer treatment. Results indicated that ethanolic extracts from TC contained phenolic and flavonoid compounds with robust antioxidant activity. TC treatments reduce CCA cell viability, inhibiting growth and inducing apoptosis in a dose-dependent manner. The Bax/Bcl-2 ratio increases, signifying a pro-apoptotic shift. Importantly, TC extract not only decreases cell viability but also augments the inhibitory effect of cisplatin in CCA cells. These results provide valuable insights into TC’s therapeutic mechanisms and its potential to synergize with conventional chemotherapeutic agents, offering a promising avenue for the development of alternative and more effective strategies for CCA treatment.

## 1. Introduction

Cholangiocarcinoma (CCA) is an aggressive malignancy of bile duct epithelial cells [1]. Notably, the northeast region of Thailand (including Khon Kaen Province) is a hotspot for *Opisthorchis viverrini* (*O*. *viverrini*) infection. Numerous studies have recognized *O*. *viverrini* infection as a major contributor to CCA carcinogenesis [2], and the International Agency for Research on Cancer (IARC) has classified *O*. *viverrini* as a Group 1 biological carcinogen in humans [3]. Repeated infection with *O*. *viverrini* triggers chronic inflammation and oxidative stress, which have been linked to oxidative stress-driven CCA development [4–6]. CCA is typically asymptomatic in its early stages, leading to late-stage diagnoses when treatment options are often ineffective, resulting in high mortality rates [7]. Currently, surgical resection remains the only definitive treatment for CCA, but survival rates post-surgery are low due to high recurrence rates [8]. Gemcitabine, either alone or in combination with cisplatin, is considered the standard first-line chemotherapeutic regimen for patients with unresectable biliary tract cancers [9, 10]. However, cisplatin is associated with adverse drug reactions, including nausea, vomiting, renal toxicity, and hypersensitivity [11]. Additionally, CCA cells frequently develop resistance to standard chemotherapies, which contributes to cancer progression, increased complications, and reduced survival times [12, 13]. Consequently, research into the use of alternative therapeutic agents (such as phytochemicals) to enhance CCA treatment efficacy is becoming increasingly urgent.

Phytochemicals are natural plant-based compounds that have been used to treat a variety of diseases, including cancer [12]. Increasing evidence support their potential to sensitize cancer cells to chemotherapeutic agents and enhance treatment efficacy [12, 14]. Many phytochemicals also possess strong antioxidant properties, helping mitigate the side effects of chemotherapy [15]. *Tacca chantrieri* André (TC), belonging to the Dioscoreaceae family and known as the “bat flower”. The plant has a unique inflorescence resembling a bat in flight and is widely used as an ornamental potted plant [16, 17]. TC is a perennial plant mainly found in southern China, Vietnam, Malaysia, and Thailand [18]. It has traditionally been used in Southeast Asia to relieve body and stomach pains, as an antidote for food poisoning, and for its analgesic, antipyretic, anti-inflammatory and insecticidal activities [18–20]. TC’s rhizome extracts contain various bioactive compounds, including diarylheptanoids, diarylheptanoid glucosides, spirostanol saponins, and withanolide glucosides [21–24]. These compounds have demonstrated anticancer properties in several studies [21, 24, 25]. For instance, spirostanol saponins, diarylheptanoid glucosides, taccalonolides and withanolides exhibit cytotoxic effects against cancer cells, [21, 24, 25]. Taccalonolides, in particular, disrupt cellular microtubules, induce mitotic arrest, and lead to cancer cell death [26]. Notably, taccalonolides also possess microtubule-stabilizing activity similar to paclitaxel, a widely used chemotherapeutic agent, and have shown the ability to overcome paclitaxel resistance in both cellular and animal models [27–29]. RCE-4, a spirostanol saponin derivative, has demonstrated potent anticancer activity by inhibiting cancer cells growth and inducing apoptosis via augmenting the Bax/Bcl-2 ratio in human cervical cancer cells [30]. In addition, several plant-derived bioactive compounds can augment the anticancer activity of cisplatin with minimal side effects. Triptolide, derived from Chinese herbal medicine *Tripterygium wilfordii* Hook F, sensitizes cisplatin-induced cytotoxicity in gastric cancer cells by enhancing apoptosis [31]. There is also evidence that saponins can enhance cisplatin’s cytotoxicity in human colon tumor cells by altering plasma membrane permeability [32]. Accumulating evidence indicates that TC’s bioactive compounds play a significant role in targeting cancer cells, and combining them with chemotherapeutic agents may offer a synergistic effect, reducing required dosages and minimizing side effects. However, the effects of TC rhizome extract on CCA cells, both alone and in combination with cisplatin, remain largely unexplored. This prompted us to hypothesize that TC may possess anticancer properties and enhance chemotherapy efficacy.

In this study, we aimed to quantify the total phenolic and flavonoid contents of TC extract, as well as assess its free radical scavenging capacity using the DPPH assay and its antioxidant activity using the FRAP assay, in CCA cell lines (KKU-213A and KKU-213C). We also examined the cytotoxic effects of TC on these cell lines, along with the underlying molecular mechanisms, using MTT assay, flow cytometry, and western blot analysis. Additionally, we investigated the potential synergistic effects of combining TC with cisplatin in these cell lines.

## 2. Materials and methods

### 2.1 Chemicals and reagents

Gallic acid, quercetin, DPPH (2,2-diphenyl-1-picrylhydrazyl), MTT (3-(4,5-dimethylthiazol-2-yl)-2,5-diphenyltetrazolium bromide) formazan and dimethyl sulfoxide (DMSO) were purchased from Sigma-Aldrich (St. Louis, MO, United States). Folin-ciocalteu’s phenol reagent was purchased from Merck KGaA (Darmstadt, Germany). Dulbecco’s Modified Eagle Medium (DMEM) medium, Fetal bovine serum, and BCA™ Protein Assay kit was procured from Thermo Fisher Scientific (Waltham, MA, USA).

### 2.2 Cell lines

Two human CCA cell lines, including KKU-213A (poorly-differentiated squamous cell carcinomas) and KKU-213C (adenosquamous carcinoma) [33] were used in this study. These *O*. *viverrini*-associated cell lines were developed at the Cholangiocarcinoma Research Institute (CARI), Khon Kaen University, and deposited in the Japanese Cancer Research Resources Bank (JCRB, Ibaraki city, Osaka, Japan). All cell lines were cultured in DMEM medium (Thermo Fisher Scientific, Waltham, MA, USA), supplemented with 10% heat-inactivated fetal bovine serum, penicillin (100 units/mL), streptomycin (100 μg/mL), and were maintained under 5% CO_2_ at 37°C in a humidified incubator. The cells were subcultured for one passage at 70–80% confluence.

### 2.3 Plant material

The plant used in this study is colloquially called bat flower, has the scientific name *Tacca chantrieri* André, and has been coded as TC. The plant authenticated was performed by Mr. Pakorn Tippayasri (Collector & Collection Number: P. Tippayasri 254), a botanist. A voucher specimen (TTM No. 0006797) has been deposited in the Thai Traditional Medicine Herbarium: TTM Herbarium, Bangkok, Thailand. The plant material was dried in a hot air oven at 60°C. The dried plant was ground to a fine powder before extraction.

### 2.4 Crude extraction and sample preparation

A 500-gram sample of TC’s rhizome extract powder from 2.3 was macerated in 1,500 mL of 95% ethanol for 7 days. Each of mixture was filtered through Whatman #2 filter paper with a chilled glass Buchner funnel and rinsed with additional ethanol 2 times. The combination ethanolic extraction was evaporated at 40°C. Then, the obtained dry extract was stored in an amber glass bottle at 2–4°C for further use.

### 2.5 Determination of total phenolic compounds

The total phenolic content of the TC extract was assessed according to a modified method of Folin-Ciocalteu [34]. TC extract was dissolved in 100% DMSO, and the final concentration was adjusted to 1 mg/mL. Briefly, 20 μl of the extract was mixed with 100 μl of Folin-Ciocalteu reagent (Merck KGaA, Darmstadt, Germany) previously diluted with distilled water at a 1:10 w/v ratio, and the mixture was placed in the dark for 30 min at room temperature. Subsequently, 80 μl of 7% sodium carbonate was added. The absorbance of the mixture was then measured at 750 nm using a microplate reader (Tecan/Sunrise Microplate Reader, Switzerland). Various concentrations of gallic acid (Sigma-Aldrich, St Louis, MO, USA) ranging from 10 to 500 μg/mL were employed as a standard. Total phenolic content was reported as micrograms of gallic acid equivalent per milligram dry weight (μg GAE/mg dry wt.), in comparison to the standard compound. The experiment was conducted in triplicate.

### 2.6 Determination of total flavonoid contents

The total flavonoid contents of TC extracts was assessed using a modified method of the aluminum chloride colorimetric method [34]. Aliquots (30 μL) of TC extracts (1 mg/mL) were mixed with 170 μL of absolute ethanol and 10 μL of 10% AlCl_3_. Subsequently, 10 μL of 1 M CH_3_CO_2_K and 30 μL of distilled water were added to the mixture and left for 30 minutes at room temperature. Finally, the absorbance at 415 nm was measured using a microplate reader (Tecan/Sunrise Microplate Reader, Switzerland). Total flavonoid contents were calculated from quercetin standard curve in the range of 10–500 μg/mL concentration which was purchased from Sigma Aldrich (St Louis, MO, USA). The data were reported as micrograms of quercetin equivalent per milligrams dry weight (μg QE/ mg dry wt.).

### 2.7 Ferric reducing antioxidant power (FRAP) assay

The FRAP assay was employed to determine the antioxidant power of TC extracts, with slight modifications [34]. In brief, the working FRAP reagent was freshly prepared by mixing 0.25 M sodium acetate buffer pH 3.6, 10 mM 2,4,6-Tripyridyltriazine (TPTZ) (Sigma-Aldrich, St Louis, MO, USA) in sodium acetate buffer, 20 mM FeCl_3_•6H_2_O (Merck KGaA, Darmstadt, Germany) at ratio of 10:1:1. Subsequently, 18 μL of TC extracts were mixed with 180 μL of FRAP reagent and left at room temperature for 30 min at 37°C. The absorbance at 593 nm was measured (Tecan/Sunrise Microplate Reader, Switzerland). A standard curve was constructed using different concentrations (ranging from 10 to 500 μg/mL). The antioxidant activity was reported in micrograms of ascorbic acid equivalent per milligrams of dry weight (μg AAE/ mg dry wt.). This assay was performed in triplicate.

### 2.8 2,2-diphenyl-1-picrylhydrazyl (DPPH) radical scavenging activity assay

The DPPH radical scavenging ability was assessed using a modified method [34]. The working reagent was prepared by dissolving DPPH in absolute ethanol at a concentration of 0.1 mM DPPH (Sigma Aldrich, St Louis, MO, USA). TC extract was diluted in 100% DMSO at concentrations of 0.05–8.00 mg/mL, respectively. Twenty microliters of samples and 180 μL DPPH solution were added to a 96-well plate and incubated at room temperature in the dark for 30 min. The absorbance was then measured at a wavelength of 517 nm using a microplate reader (Tecan/Sunrise Microplate Reader, Switzerland). The DPPH free radical scavenging capacity was determined and compared with the standard ascorbic acid. The percentage of radical scavenging activity was calculated using the following equation:

$$\text{Percentage}\;\text{of}\;\text{scavenging}\;\text{effect}\left(\%\right)=100\times \left[\left(\text{Ab}–\text{As}\right)/\text{Ab}\right]$$

Where Ab represents the absorbance of reagent bank, and As is the absorbance of the reaction with the extracts.

### 2.9 Cell viability assay using MTT

The optimal crude extract concentration was determined to be in the range of 1–50 μg/mL. We used this range, increasing the concentration by approximately 2.5 to 5 μg/mL increments. For cell cytotoxicity of TC, cells (2 x 10^3^ cells per well) were seeded into 96-well plates in triplicate. After 24 h, the cells were treated with media containing various concentrations of TC crude extracts (0, 5, 7.5, 10, 12.5, 15, 20 and 25 μg/mL) for 24, 48, and 72 h. The control group was 100% cell viability. Inhibitory concentration (IC) including IC_25_ and IC_50_ valves were presented. For the combination treatment of TC and cisplatin, cells (2 x 10^3^ cells per well) were seeded into 96-well plates. After 24 h, the cells were treated with media containing various concentrations of TC crude extracts and cisplatin concentrations at 5, 10 and 20 μM for 24 h.

At the end of each treatment, the viability of cells was determined using the MTT assay. Briefly, 100 μl of 0.5 mg/mL MTT solution was added to each well and incubated at 37°C for 4 h in the dark. Subsequently, the MTT solution was then aspirated and replaced with 100 μl of DMSO to dissolve the precipitate dark blue crystals. The absorbance was measured at 540 nm using a microplate reader (Tecan/Sunrise Microplate Reader, Switzerland).

### 2.10 Detection of cell apoptosis by flow cytometry

Cell apoptosis was investigated using the Annexin V FITC apoptosis detection kit (AD10-10) (Dojindo Europe GmbH, Munich, Germany). Cell lines (100,000 cells/well) were seeded into 6-well plates and treated with various concentrations of TC (KKU-213A: 13, 15, 17 μg/ml and KKU-213C: 13, 14, 15 μg/ml). After 24 h, trypsinized cells were washed with 1× PBS. Annexin V, FITC and propidium iodide (PI) were added and incubated at room temperature for 10 min in a dark area. Finally, stained cells were examined using a flow cytometer (BD FACSCanto™ II, BD Biosciences, CA, USA) and analyzed with FlowJo 10 software.

### 2.11 Western blot analysis

Total protein was extracted using RIPA lysis buffer (0.5 M Tris-HCl pH 7.4, 150 mM NaCl, 1% (v/v) Tween-20, 1% (w/v) sodium deoxycholate, 0.1% (w/v) SDS) containing a cocktail of protease and phosphatase inhibitors. BCA™ Protein Assay kit (Pierce Biotechnology, Rockford, IL, USA) was used to determine protein concentration. Protein extracts (20 μg) were separated using sodium dodecyl sulfate polyacrylamide gel electrophoresis (SDS-PAGE), then transferred onto PVDF membranes (EMD Millipore, Billerica, MA, USA). Non-specific binding was blocked by 5% skimmed milk, then membranes were probed with specific primary antibodies (rabbit polyclonal anti-Bax antibody, rabbit polyclonal anti-Bcl-2 antibody, and mouse monoclonal anti-β-actin antibody) (Proteintech, Illinois, USA) for 1 h at room temperature followed by 4°C with gentle shaking overnight. After washing, secondary antibody (Invitrogen™, USA) was added, membranes were then exposed to ECL Prime Western Blotting Detection Reagent (Amersham, Cytiva Life Sciences, UK). β-actin antibody (Sigma Aldrich, St Louis, MO, USA) was used as an internal loading control. The band intensity was examined by the Amersham ImageQuant 800 (Amersham, Cytiva Life Sciences, UK).

### 2.12 Statistical analysis

The data were analyzed from at least three independent experiments using GraphPad Prism 8.0 software (GraphPad. Software Inc.). Total phenolic, flavonoid and antioxidant by FRAP assay were represented as mean ± standard deviation (S.D.). Antioxidant by DPPH assay was presented by 50% effective concentration (EC_50_). Half maximal inhibitory concentration (IC_50_) of cell treatment was presented as mean ± SD. Analysis of variance (ANOVA) was used for cell viability, apoptosis, and protein expression. *p* < 0.05 was considered to indicate statistical significance.

## 3. Results

### 3.1 Phenolic and flavonoid contents and antioxidant activity assessments

**Fig 1:**
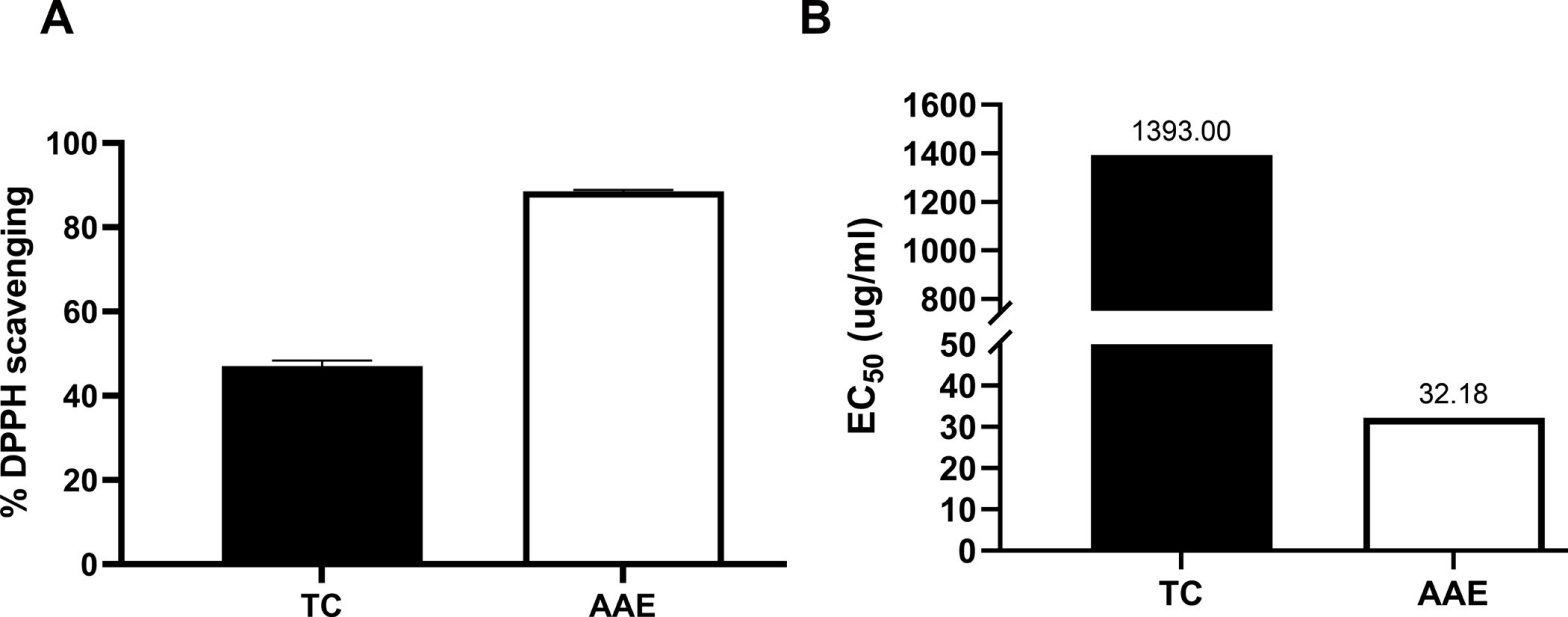
DPPH scavenging activity of *Tacca chantrieri* André (TC) extract. DPPH scavenging activity of TC extract and ascorbic acid (AAE) at 1 mg/mL (A), EC_50_ values of TC extract and AAE (B). Data are presented as the mean ± standard deviation of triplicate experiments.

To determine the bioactive compounds in TC extract, we quantified total phenolics and flavonoids using the Folin-Ciocalteu and aluminum chloride methods, respectively. TC extract contained 84.243 ± 1.904 μg GAE/mg dry wt. of total phenolics and 10.738 ± 0.997 μg quercetin/mg dry wt. of flavonoids (Table 1). We assessed antioxidant activity using the DPPH and FRAP assays. In the DPPH assay, TC extract (1 mg/mL) exhibited 47.038% DPPH scavenging with an EC_50_ of 1393.00 μg/mL (Fig 1). The FRAP assay indicated a ferric reducing power of 95.044 ± 3.769 μg AAE/mg dry wt. (Table 1). These findings suggest that TC rhizome extract contains bioactive compounds with potent antioxidant activity, which may have potential to reduce or neutralize oxidative stress in CCA cells.

**Table 1:** Phenolic, flavonoid contents and antioxidant activity of TC extracts.

Extract	Total phenolic content (μg GAE/mg dry wt.)	Total flavonoid content (μg quercetin/mg dry wt.)	FRAP assay (μg AAE/mg dry wt.)
TC	84.243 ± 1.904	10.738 ± 0.997	95.044 ± 3.769

TC: *Tacca chantrieri* André, GAE: gallic acid equivalent, AAE: ascorbic acid equivalent, mg dry wt: milligram dry weight.

### 3.2 TC extract inhibits CCA cell viability

**Fig 2:**
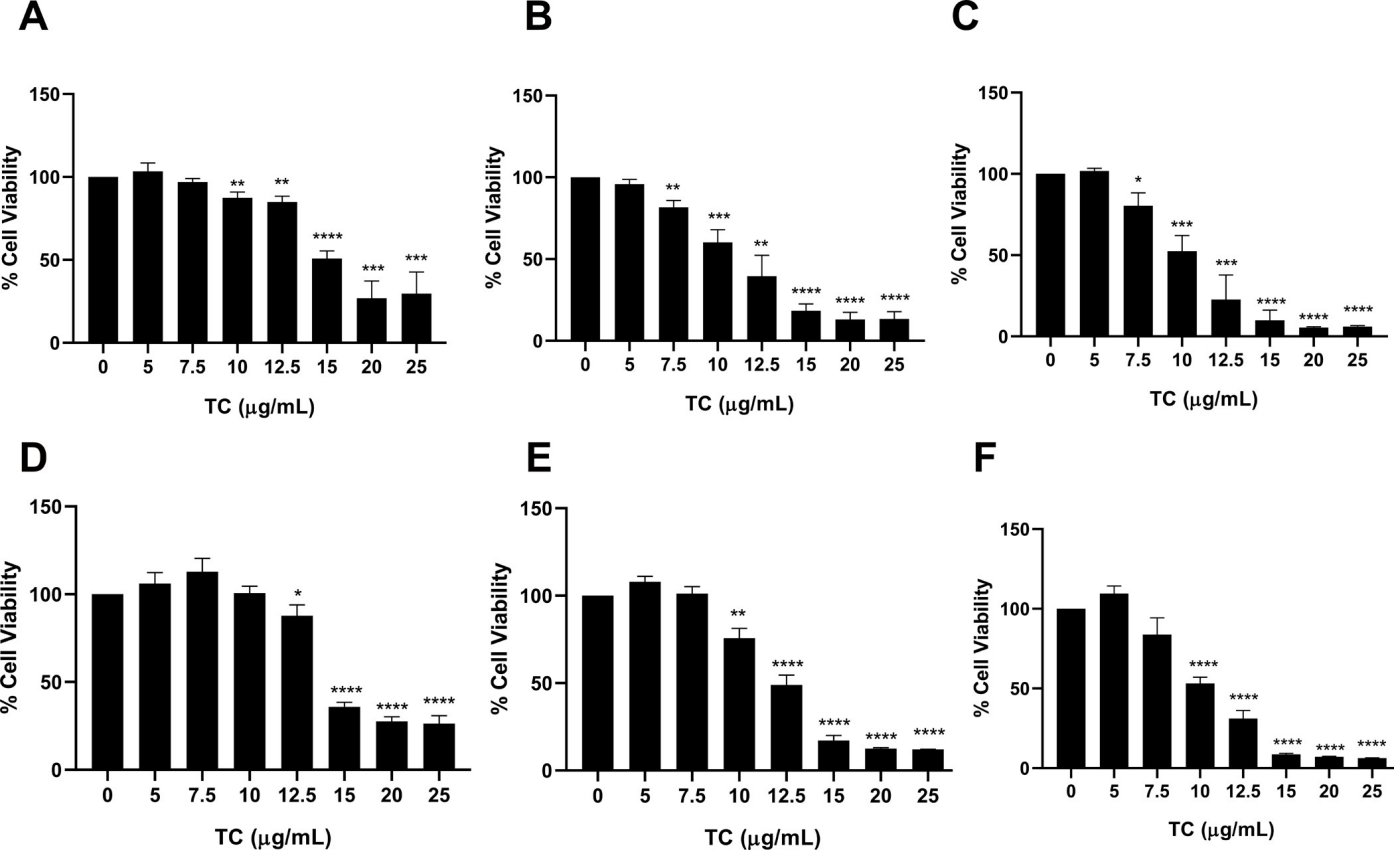
Anti-cancer effect of TC extract on CCA cell lines. Fig. A-C show KKU-213A at 24 h (A), 48 h (B) and 72 h (C). Fig D-F show KKU-213C at 24 h (D), 48 h (E) and 72 h (F). Data are presented as the mean ± standard deviation of triplicate experiments. (*p < 0.05, **p < 0.01, ***p < 0.001, and ****p < 0.0001) compared to control group.

To evaluate the effect of TC on CCA cells, KKU-213A and KKU-213C were treated with varying concentrations of TC extract (0, 5, 7.5, 10, 12.5, 15, 20 and 25 μg/mL) for 24, 48, and 72 h. using the MTT assay. In KKU-213A cells, TC at concentration of 10, 12.5, 15, 20 and 25 μg/mL significantly reduced cell viability after 24 h (Fig 2A). At 48 h and 72 h, a significantly reduced in cell viability was observed at concentration of 7.5, 10, 12.5, 15, 20 and 25 μg/mL (Fig 2B and 2C).

Similarly, in KKU-213C cells, TC concentrations of 12.5, 15, 20 and 25 μg/mL significantly reduced cell viability at 24 h (Fig 2D), while cell viability was significantly reduced at concentration 10, 12.5, 15, 20 and 25 μg/mL at 48 h and 72 h (Fig 2E and 2F). These findings suggest that TC extract reduces the viability of CCA cells in a dose- and time-dependent manner. Additionally, IC_25_ and IC_50_ were examined and shown in Table 2. For KKU-213A, IC_25_ and IC_50_ were 13.2 ± 0.2, 15.3 ± 0.4 μg/mL at 24 h, 8.5 ± 0.5, 11.5 ± 1.2 μg/mL at 48 h, and 8.1 ± 0.8, 10.4 ± 0.9 μg/mL at 72 h. For KKU-213C, IC_25_ and IC_50_ were 13.2 ± 0.2, 14.3 ± 0.2 μg/mL at 24 h, 10.1 ± 0.3, 12.4 ± 0.3 μg/mL at 48 h, and 7.9 ± 0.5, 10.3 ± 0.1 μg/mL at 72 h. Therefore, the IC_25_, IC_50_ values for TC treatment in both CCA cell lines were selected for further investigated.

**Table 2:** Inhibitory concentration (IC), IC_25_ and IC_50_ of TC-treated CCA cells.

Cell lines	Times	TC (μg/mL) ± SD	
		IC_25_	IC_50_
**KKU-213A**	**24h**	13.2 ± 0.2	15.3 ± 0.4
	**48h**	8.5 ± 0.5	11.5 ± 1.2
	**72h**	8.1 ± 0.8	10.4 ± 0.9
**KKU-213C**	**24h**	13.2 ± 0.2	14.3 ± 0.2
	**48h**	10.1 ± 0.3	12.4 ± 0.3
	**72h**	7.9 ± 0.5	10.3 ± 0.1

### 3.3 TC extract induces CCA cell death via apoptosis pathway

**Fig 3:**
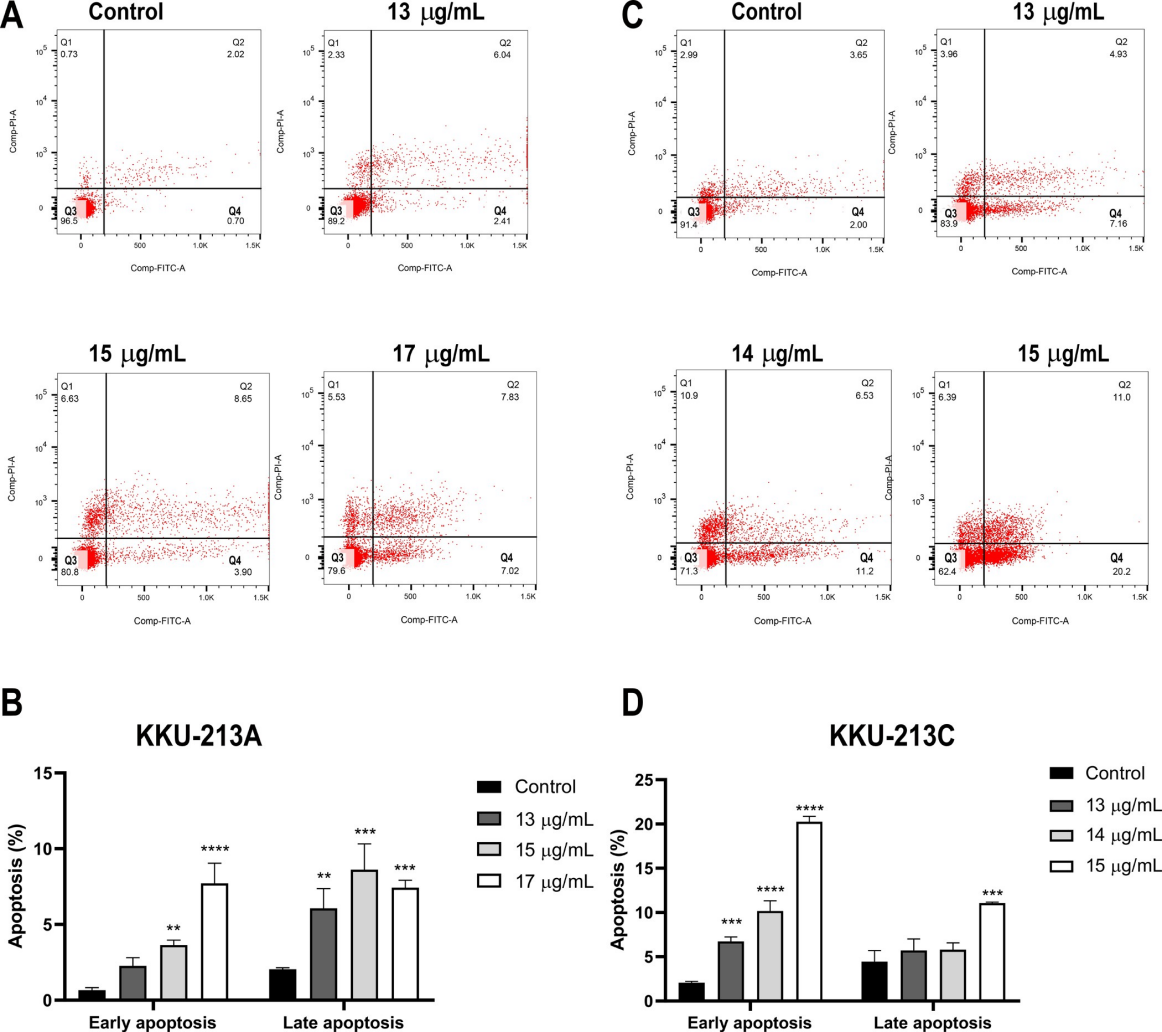
Apoptosis assay using flow cytometry following staining with Annexin V, FITC and PI. (A) Scatter plot of apoptotic cells in KKU-213A. (B) Percentage of apoptotic cells in early and late apoptosis in KKU-213A. (C) Scatter plot of apoptotic cells in KKU-213C. (D) Percentage of apoptotic cells in early and late apoptosis in KKU-213C. Data are presented as the mean ± standard deviation of triplicate experiments. (*p < 0.05, **p < 0.01, ***p < 0.001, and ****p < 0.0001) compared to control group.

**Fig 4:**
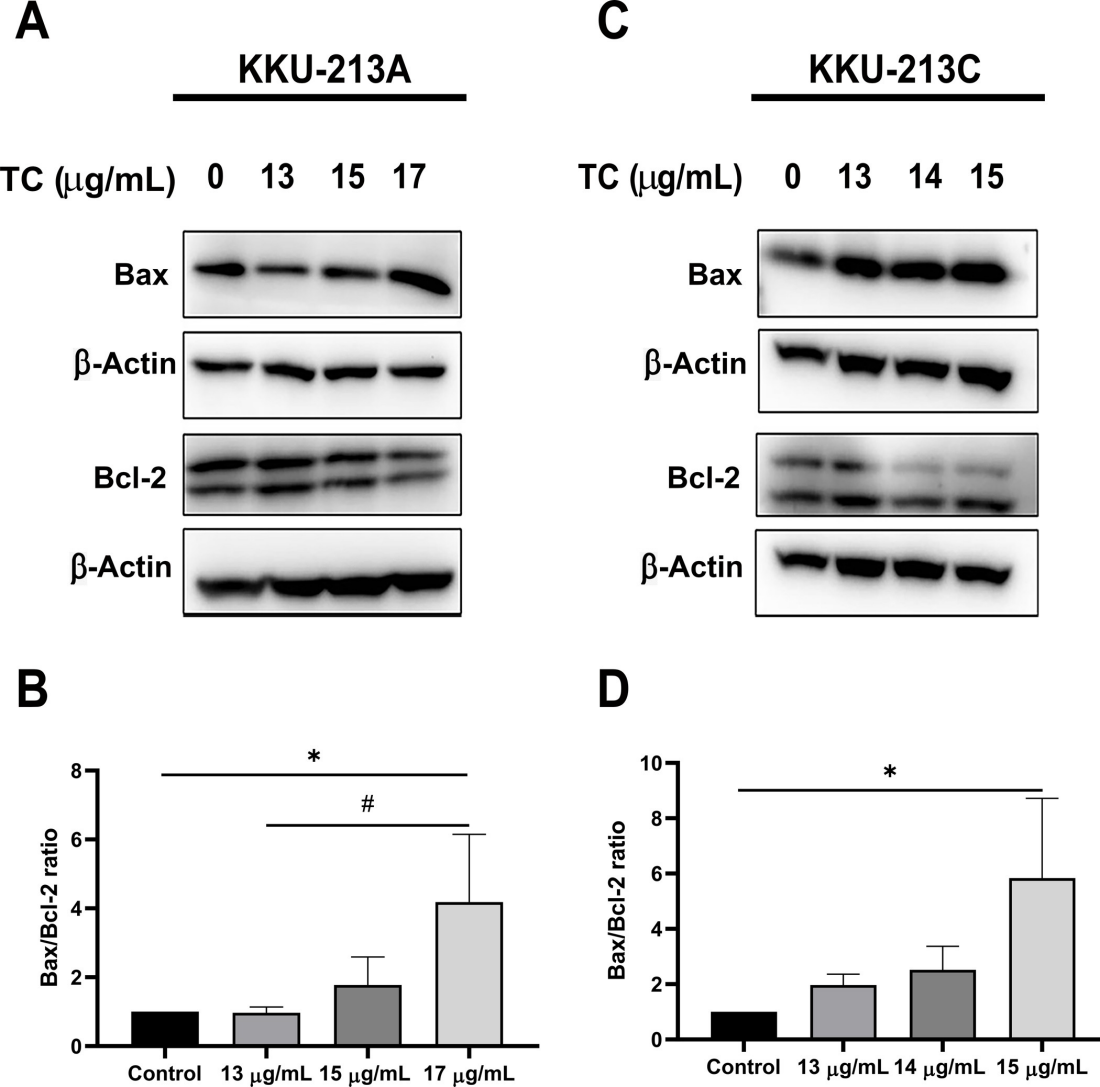
Western blot analysis in TC-treated CCA cell lines. Representative picture of Bax and Bcl-2 expression in KKU-213A (A) and KKU-213C (C). The graphical plots demonstrate the relative intensity of the Bax/Bcl-2 ratio in KKU-213A (B) and KKU-213C (D). Data are presented as the mean ± standard deviation of triplicate experiments. (*p < 0.05 compared to control group, ^#^*p* < 0.05 compared to 13 μg/mL).

To investigate the efficiency of TC extract on the induction of apoptosis, CCA cell lines were treated with various concentrations of TC (IC_25_, IC_50_, and more than IC_50_) at 24 h. After harvest, cell apoptosis was examined using flow cytometry. After 24 h of treatment, a significant increase in apoptotic cells of both early and late apoptosis was observed in KKU-213A with a greater concentration of TC, particularly at 15 μg/mL (IC_50_) and 17 μg/mL (more than IC_50_) (Fig 3A and 3B). In KKU-213C, the number of apoptotic cells in early apoptosis was significantly increased in all concentrations including 13 μg/mL (IC_25_), 14 μg/mL (IC_50_) and 15 μg/mL (more than IC_50_), while a significant increase in apoptotic cells in late apoptosis was found in 15 μg/mL (more than IC_50_) of TC (Fig 3C and 3D). Notably, a significant increase in the population of early and late apoptotic cells was observed in both CCA cell lines after 24 h of treatment with TC extract in a dose-dependent manner. Western blot analysis was performed to assess the expression of apoptosis-related proteins, including Bax and Bcl-2. The results showed a significant increase in the Bax/Bcl-2 ratio in KKU-213A cells treated with 17 μg/mL of TC and in KKU-213C cells treated with 15 μg/mL of TC (Fig 4A–4D).

### 3.4 TC extract combined with cisplatin enhances inhibitory effects on CCA cells

**Fig 5:**
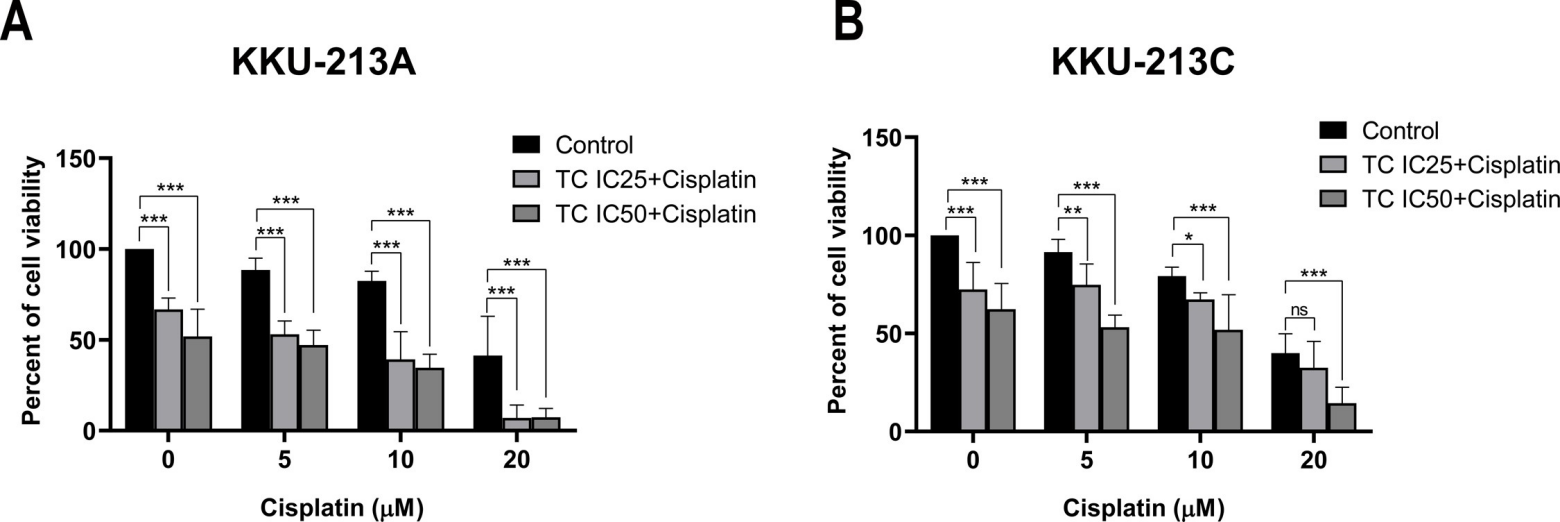
Anti-cancer effects of combined TC extract and cisplatin. KKU-213A and KKU-213C cells were treated with cisplatin at concentrations 0, 5, 10 and 20 mg/ml for 24 h and concentrations at IC_25_ and IC_50_ of TC. Data are presented as the mean ± standard deviation of five replicate experiments. (*p < 0.05, **p < 0.01, and ***p < 0.001) compared to control group.

To investigate the effect of TC extract in combination with chemotherapy, CCA cell lines were treated with various concentrations of cisplatin (0, 5, 10 and 20 μM). Each concentration was combined with low concentration TC extract (IC_25_ and IC_50_). Treatment using TC at IC_25_ and IC_50_ significantly inhibited cell viability in both CCA cell lines when compared to controls (Fig 5A and 5B). Moreover, combined treatments of TC at IC_25_ and IC_50_ with 5, 10 and 20 μM of cisplatin at 24 h significantly decreased CCA cell viability in KKU-213A and KKU-213C. However, there was no significant difference with a combination treatment of TC at IC_25_ with cisplatin at 20 μM in KKU-213C (Fig 5A and 5B). When considering IC_50_, we found that the IC_50_ of cisplatin alone was 24.0±15.5 μM in KKU-213A and 23.0±4.4 μM in KKU-213C. In the combined treatment of IC_50_ of cisplatin at with IC_25_ and IC_50_ of TC in KKU-213A showed the IC_50_ values of cisplatin in combination group with TC at concentration IC_25_ and IC_50_ (5.0±1.8 μM and 4.2±2.0 μM, respectively) have lower than cisplatin alone as shown in Table 3. Similar to KKU-213A, the IC_50_ values of cisplatin less than cisplatin alone in KKU-213C after being combination of cisplatin and TC at concentration IC_25_ and IC_50_ (14.3±4.7 μM and 6.6±2.0 μM, respectively). Suggesting that the combination of TC extract and cisplatin significantly reduced the inhibitory concentration required to kill 50% of both CCA cells compared to cisplatin alone. Thus, this finding indicated that TC extract not only decreases cell viability but also enhances the inhibitory effect of cisplatin in CCA cells.

**Table 3:** The half-maximal inhibitory concentration (IC_50_) values of combination treatment between cisplatin and TC extract.

CCA cells	IC_50_ (μM) ±SD		
	Cisplatin	TC at IC_25_+Cisplatin	TC at IC_50_+Cisplatin
**KKU-213A**	24.0 ± 15.5	5.0 ± 1.8	4.2 ± 2.0
**KKU-213C**	23.0 ± 4.4	14.3 ± 4.7	6.6 ± 2.0

## 4. Discussion

Phytochemicals are natural plant-based compounds, that have long been a source of drug development, including cancer treatments, such as paclitaxel and vinblastine. Paclitaxel, derived from the bark of *Taxus brevifolia*, is widely used to treat various cancers, including ovarian, esophageal, breast, lung, and pancreatic cancers [35]. In addition to their role in cancer therapy, many plant extracts support health and wellness through their antioxidant activity, because the imbalance of cellular redox homeostasis contributes to many diseases including cancer [36]. Chemotherapy often increases oxidative stress in both cancerous and normal cells, leading to severe side effects [37]. CCA is believed to be driven by oxidative stress, particularly from chronic inflammation linked to liver fluke infection [5]. Elevated oxidative stress markers, such as 8-oxodG, have been associated with poor prognosis in CCA patients, further suggesting the role of oxidative stress in cancer promotion and progression [38, 39], In light of this, our study aimed to assess the antioxidant potential of TC extract to counter oxidative stress. The extract was found to be rich in phytochemicals, particularly flavonoids and phenolic compounds. Even though, the different levels of these compounds were observed when compared with the pervious study [40]. This might be the effect of using different parts of the plant, which can affect the levels of these compounds [41]. To confirm the antioxidant properties of TC extract, we performed the DPPH radical scavenging and FRAP assay. The DPPH assay evaluates a compound’s ability to donate an electron to neutralize free radicals, while the FRAP assay measures its ability to reduce ferric (Fe^3+^) to ferrous (Fe^2+^) ions [42, 43]. Our results showed that TC extract possesses antioxidant activity in both assays, supporting previous studies that attributed this effect to bioactive compounds like saponins, which have been reported to reduce nitric oxide (NO)-related inflammation [44]. Additionally, TC extract is known to contain compounds with analgesic, antipyretic, and anti-inflammatory properties,[20], as well as neuroprotective effects against cognitive impairment and neuroinflammation [45]. Thus, TC rhizome extract is rich in compounds with potent antioxidant activity, making it a promising candidate for reducing oxidative stress and inflammation. This could help mitigate the adverse effects of chemotherapy and offer additional health benefits.

Treating CCA is highly challenging due to the late-stage diagnosis in most cases. Surgery is the only curative option, while chemotherapy is often ineffective [46]. Consequently, CCA patients experience high mortality rates and a significant risk of cancer recurrence [47]. Moreover, the adverse side effects of chemotherapy contribute to patient suffering, highlighting the urgent need for more effective therapeutic agents. The rhizomes of various *Tacca* species have long been used in traditional medicine and are known to contain bioactive compounds with anticancer properties [21, 25]. For instance, evelynin, a compound isolated from TC rhizomes, exhibits potent cytotoxicity against several cancer types, including melanoma, breast, prostate, and cervical carcinoma, with IC_50_ values of 4.1, 3.9, 4.7, and 6.3 μM, respectively [48]. Taccachatrones A-G have also shown strong cytotoxicity against various human cancer cell lines [49]. Another group of compounds, taccalonolides, display significant anticancer effects by targeting cellular microtubules, leading to cancer cell death. Interestingly, taccalonolides have been identified as a class of microtubule-stabilizing agents, similar to the chemotherapeutic drug paclitaxel, and have shown the ability to overcome paclitaxel resistance, making them a promising candidate for treating drug-resistant cancers [26–29]. These findings suggest that the rhizome of TC contains bioactive compounds with considerable cytotoxic potential, offering a basic for future anticancer therapy development [48, 49]. In this study, the cytotoxic effects of TC rhizome extract were evaluated on CCA cell lines (KKU-213A and KKU-213C) using the MTT assay. The extract exhibited strong antiproliferative effects on CCA cells in a dose- and time-dependent manner. Slight variations in IC_25_ and IC_50_ values were observed between KKU-213A and KKU-213C cell lines, likely due to characteristic differences between cell lines. The cytotoxicity of TC extract was also evaluated in the immortalized human cholangiocyte cell line MMNK1 [50]. The results showed that the IC_50_ values for MMNK1 cells were higher than those for CCA cells following TC extract treatment at 24, 48, and 72 h, with IC_25_ and IC_50_ values of 16.50 ± 1.3 and 23.79 ± 3.4 μg/mL at 24 h, 12.81 ± 2.2 and 15.21 ± 2.0 μg/mL at 48 h, and 12.49 ± 2.1 and 13.59 ± 1.4 μg/mL at 72 h, respectively. These results suggest a lower toxicity to normal cholangiocyte cells (S1 Fig and S1 Table). Our findings indicate that TC extract may exert anticancer activity against CCA cells while minimizing toxicity to normal cells, offering a promising avenue for developing safer and more effective cancer therapies.

Programmed cell death, or apoptosis, is triggered by various physiological stressors that cancer cells encounter during tumorigenesis or anticancer therapy. However, cancer cells often develop mechanisms to evade apoptosis, contributing to their resistance to cell death, a hallmark of cancer [51]. A key regulator of apoptosis is the balance between two proteins Bax and Bcl-2. An increase in the Bax/Bcl-2 ratio shift the cell towards apoptosis, promoting cell death [52]. Unfortunately, in CCA, the upregulation of certain Bcl-2 family members contributing to chemotherapeutic resistance [53]. Interestingly, our results demonstrate that TC extract induces early and late apoptosis in CCA cell lines by upregulating the pro-apoptotic protein Bax and downregulating the anti-apoptotic protein Bcl-2. Additionally, the bioactive compounds in TC, including spirostanol saponins and diarylheptanoids, may promote apoptosis by regulating the expression of apoptosis-related proteins. Previously, a spirostanol saponin derivative has been shown to inhibit cancer cell growth and induce apoptosis by increasing the Bax/Bcl-2 ratio in human cervical cancer cells [30, 44]. Moreover, diarylheptanoids from *Alpinia officinarum* have been found to inhibit neuroblastoma cell proliferation by inducing cell cycle arrest and apoptosis [54]. Diarylheptanoids from the rhizome of ginger (*Zingiber officinale*) also exhibit significant antiproliferative activity on HL-60 cells by promoting apoptosis [55]. These studies highlight the potential of TC extract as a therapeutic agent for enhancing apoptosis in CCA cells, possibly through the induction of the Bax/Bcl-2 ratio by bioactive compounds such as spirostanol saponins and diarylheptanoids.

Cisplatin (cis-diamminedichloroplatinum II) is a widely used platinum-based chemotherapy agent for treating various cancers, including CCA [56]. It actions by crosslinking with purine bases on DNA, disrupting repair mechanisms, causing DNA damage, and ultimately triggering apoptosis in cancer cells [56, 57]. However, the increasing incidence of cisplatin resistance remains a significant clinical challenge, mainly driven by mechanisms such as impaired apoptotic signaling pathways [58]. Studies have identified the overexpression of the Bcl-2 protein as a key factor contributing to cisplatin resistance in human bladder cancer cells [59]. Extensive research has revealed that plant-derived phytochemicals can enhance the efficacy of chemotherapeutic drugs and help overcome chemoresistance. For instance, the diarylheptanoid hirsutenone, found in *Alnus hirsuta* bark, sensitivity cisplatin-resistant ovarian cancer cells to cisplatin by activating p53-dependent apoptosis [60]. Similarly, shikonin, a compound from the Chinese herb *Lithospermum erythrorhizon*, enhances cisplatin’s anticancer effects in colon cancer cells by promoting apoptosis [61]. Interestingly, saponins have shown potential in overcoming chemoresistance by increasing cisplatin uptake via modulation of membrane permeability in human colon cancer cells [32]. Hence, combining cisplatin with phytochemicals has been considered a promising strategy to combat drug resistance and reduce adverse effects. Our results demonstrated that the combined treatment of TC extract with cisplatin induced greater cell death compared to cisplatin alone. This was evidenced by a lower IC_50_ in both CCA cell lines when treated with the combination in a dose-dependent manner. These findings, along with previous studies, suggest that TC may contain bioactive compounds that not only exhibit anticancer properties but also enhance cisplatin’s inhibitory effects in CCA cells, suggesting a synergistic effect. While the precise mechanisms remain unclear, based on our findings and previous reports suggest that TC extract may enhance the efficacy of cisplatin in CCA cells through two primary mechanisms, (i) modulating the Bcl-2 family expression to increase the Bax/Bcl-2 ratio, thus restoring cisplatin sensitivity, and (ii) facilitating greater cisplatin uptake.

Taken together, our findings demonstrate that TC rhizome crude extract possessed both anticancer and antioxidant properties. The bioactive compounds within this extract enhance cytotoxicity, induce cancer cell death, and potentiate cisplatin sensitivity in CCA cells. This suggests a potential synergistic therapeutic effect, allowing for lower doses of cisplatin while maintaining its efficacy. Moreover, TC extract may overcome chemoresistance by targeting multiple cellular pathways *e*.*g*. apoptosis pathway. Further studies are necessary to elucidate the molecular mechanisms by which TC enhances cisplatin sensitivity and to identify the specific bioactive compounds present in TC extract. Additionally, it is important to evaluate TC’s effects on a broader spectrum of cancer cells, including drug-resistant lines. To fully understand TC’s therapeutic potential in combination with cisplatin, *in vivo* models and clinical trials should be conducted to validate these findings.

## Supporting information

S1 Fig Anti-cancer effect of TC extract on MMNK1 cell line. Fig. A-C show MMNK1 at 24 h (A), 48 h (B) and 72 h (C). MMNK1 treated with TC extract with a series concentration 0–25 μg/ml. The asterisk indicates statistical significance at *p < 0.05, **p < 0.01, ***p < 0.001, and ****p < 0.0001 compared to control group.
(TIF)

S1 Raw data Raw data for phenolic, flavonoid contents and antioxidant activity of TC extracts, and anti-cancer effect of TC extract on CCA cell lines. (XLSX)

S1 Raw image The original images of western blot analysis. Original western blot images showing Bax and Bcl-2 expression in KKU-213A cells treated with TC extract at 0, 13, 15, and 17 μg/ml and in KKU-213C cells treated with TC extract at 0, 13, 14, and 15 μg/ml.
(TIF)

S1 Table Inhibitory concentration (IC), IC_25_ and IC_50_ of TC-treated MMNK1 cells. The table shows IC_25_ and IC_50_ of TC-treated MMNK1 cells at different times (24, 48, and 72 h).
(DOCX)
