# Supplementary figures and images for: Antioxidative and anticancer effects of Tacca chantrieri extract enhancing cisplatin sensitivity in cholangiocarcinoma cells
Source: PLoS One. 2025 Jan 16;20(1):e0317111. doi: 10.1371/journal.pone.0317111 (PMC11737735; doi:10.1371/journal.pone.0317111)

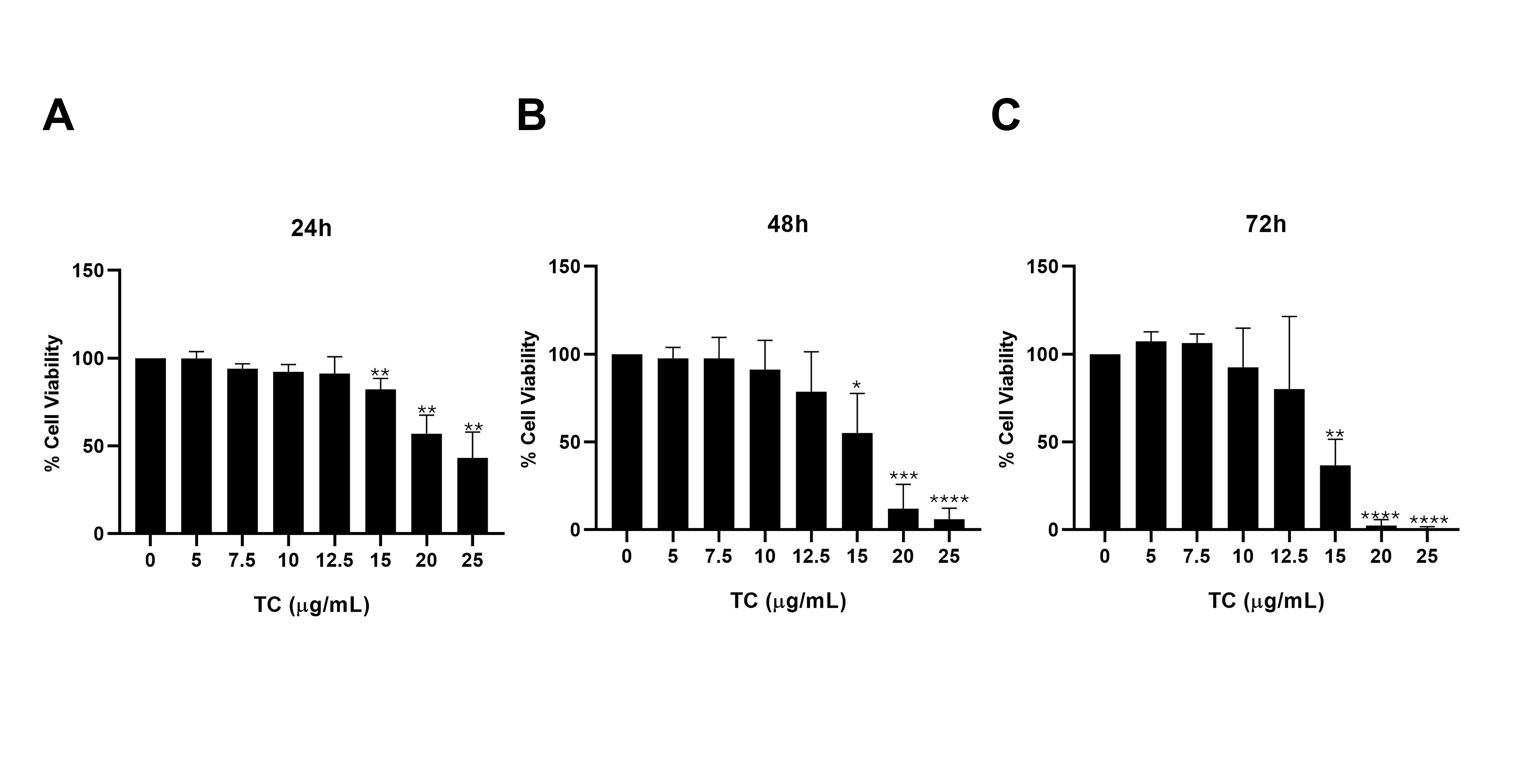

Supplement: S1 Fig — Fig. A-C show MMNK1 at 24 h (A), 48 h (B) and 72 h (C). MMNK1 treated with TC extract with a series concentration 0–25 μg/ml. The asterisk indicates statistical significance at *p < 0.05, **p < 0.01, ***p < 0.001, and ****p < 0.0001 compared to control group. (TIF) [file pone.0317111.s001.tif]

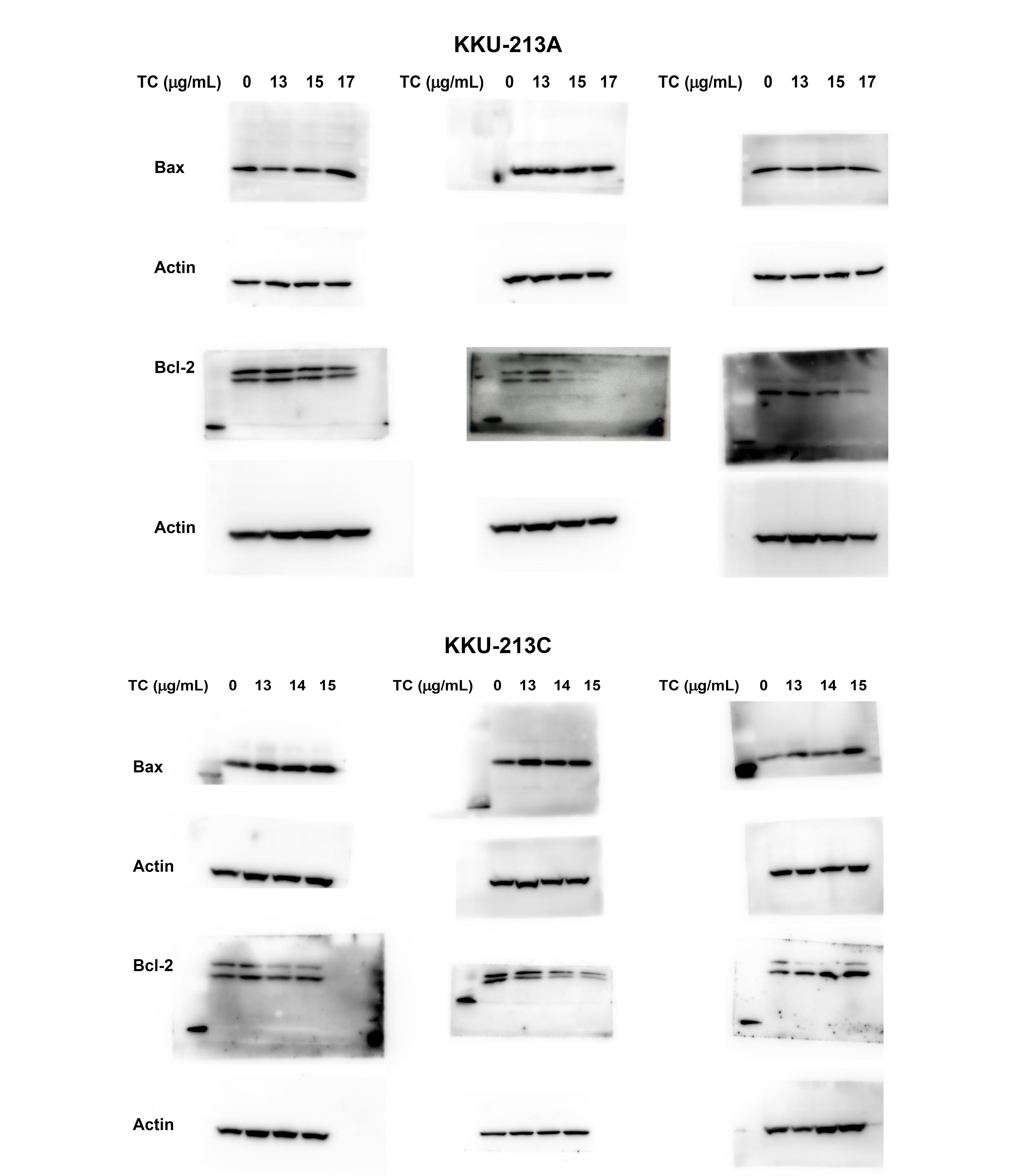

Supplement: S1 Raw image — Original western blot images showing Bax and Bcl-2 expression in KKU-213A cells treated with TC extract at 0, 13, 15, and 17 μg/ml and in KKU-213C cells treated with TC extract at 0, 13, 14, and 15 μg/ml. (TIF) [file pone.0317111.s003.tif]
