# Supplementary material for: Antioxidative and anticancer effects of Tacca chantrieri extract enhancing cisplatin sensitivity in cholangiocarcinoma cells
Source: PLoS One. 2025 Jan 16;20(1):e0317111. doi: 10.1371/journal.pone.0317111 (PMC11737735; doi:10.1371/journal.pone.0317111)
Supplement: S1 Table — The table shows IC25 and IC50 of TC-treated MMNK1 cells at different times (24, 48, and 72 h). (DOCX) [file pone.0317111.s004.docx]

**S1 Table. Inhibitory concentration (IC), IC_25_ and IC_50_ of TC-treated MMNK1 cells.**

| **Cell lines** | **Times** | **TC (μg/mL) ± SD** | |
| --- | --- | --- | --- |
|  |  | **IC_25_** | **IC_50_** |
| **MMNK1** | **24h** | 16.50 ± 1.3 | 23.79 ± 3.4 |
|  | **48h** | 12.81 ± 2.2 | 15.21 ± 2.0 |
|  | **72h** | 12.49 ± 2.1 | 13.59 ± 1.4 |
